# Prognostic Factors for the Work Participation of Sick-Listed Unemployed and Temporary Agency Workers with Psychological Problems

**DOI:** 10.1007/s10926-012-9358-0

**Published:** 2012-04-06

**Authors:** Selwin S. Audhoe, Jan L. Hoving, Karen Nieuwenhuijsen, Rafiq Friperson, Philip R. de Jong, Judith K. Sluiter, Monique H. W. Frings-Dresen

**Affiliations:** 1 Coronel Institute of Occupational Health/Research Center for Insurance Medicine, Academic Medical Center, University of Amsterdam, P.O. BOX 22700, 1100 DE Amsterdam, The Netherlands; 2 Research Institute APE, The Hague, The Netherlands

**Keywords:** Unemployment, Participation, Psychological problems, Prognostic factors, Vocational rehabilitation

## Abstract

*Introduction* Among the working population, unemployed and temporary agency workers are a particularly vulnerable group, at risk for sickness absence due to psychological problems. Knowledge of prognostic factors for work participation could help identify sick-listed workers with a high-risk for work disability and provide input for sickness absence counseling. The purpose of this study was to identify prognostic factors for the work participation of medium- and long-term sick-listed unemployed and temporary agency workers with psychological problems. *Methods* A cohort of 932 sick-listed unemployed and temporary agency workers with psychological problems was followed for one and a half years. Data collection was conducted at three time-frames: 10 months, 18 months and 27 months after reporting sick. Univariate and multiple logistic regression analyses were performed. *Results* Perceived health, full return-to-work (RTW) expectations, age and work status at 18 months were strong prognostic factors for work participation at subsequent time-frames in the univariate analyses. Multiple logistic regression revealed that full RTW expectation was a prognostic factor for future work participation in both the medium- and long-term, whereas moderate-to-good perceived health was a prognostic factor for work participation in the medium-term. Being under 45 years of age and having a positive work status at 18 months were prognostic factors for work participation in the long-term.* Conclusions* Workers’ self-appraisal of health, age and work status were strong prognostic factors for the future work participation of sick-listed unemployed and temporary agency workers with psychological problems. These findings could help occupational and insurance physicians identify high-risk sick-listed workers for sickness absence counseling.

## Introduction

In recent decades, psychological problems have been a growing cause of sickness absence [1, 2], and have emerged as a major public and occupational health problem in many countries [3]. Psychological problems are now the leading cause of sickness absence in most high-income countries, accounting for approximately 40% of the total time covered by sick notes [4]. In Europe, most of this sickness absence is caused by mild psychological problems [5, 6]. In Great Britain, approximately 40 million workdays are lost annually due to mild psychological problems [7]. Furthermore, psychological problems are strongly associated with prolonged work disability [8–10]. In The Netherlands, psychological problems account for one-third of all disability benefits [11].

Workers whose employment contract is missing (i.e., workers without an employment contract or unemployed workers) or is flexible (i.e., workers with flexible labor market arrangements, such as temporary agency workers and fixed-term contract workers) are at even greater risk for work disability due to psychological problems than the general working population as there is no employer to return to when sick-listed. Therefore workers without an employment contract or with flexible contracts are a vulnerable group. Considering the consequences of sickness absence due to psychological problems for both individuals and society, predicting which people with psychological problems are at risk for prolonged work disability is important and even more for the aforementioned vulnerable group. The identification of prognostic factors could help provide input for sickness absence counseling or interventions at an early stage to prevent long-term sick leave and the subsequent transition to permanent disability. There is some evidence that vocational interventions can improve work participation and reduce mental distress in the unemployed [12]. However, evidence about prognostic factors for the work participation of unemployed and temporary workers who are sick-listed due to psychological problems is scarce. Therefore, this research aimed to evaluate the prognostic factors for the work participation of this group. For the remainder of this article, “temporary agency workers” also refers to other flexible workers with expired fixed-term contracts.

As shown in the literature, sickness absence due to psychological problems is not determined by psychological problems alone; it is also influenced by other factors, such as work related factors, health expectations (e.g., recovery expectations) and personal factors (e.g., education level) [13, 14]. There is strong evidence that older age (>50 years) is a negative predictor for return to work (RTW) and is associated with continuing disability. Furthermore, there is limited evidence for the association of personal factors (gender, education, history of previous sickness absence, negative recovery expectations, socio-economic status) and work-related factors (e.g., the quality and continuity of occupational care) with RTW and disability [14]. However, these factors were studied in employed workers and during their first year of sickness absence. The question is whether these factors or other unknown factors apply to unemployed or temporary agency workers who have been sick for 1 year or more, because prognostic factors can change or become less or more relevant during the period of sickness absence.

Some of the above-mentioned prognostic factors for RTW, such as age, education and history of previous sickness absence, will not change during the sickness absence period. However, other factors may change or become less or more relevant during the sickness absence period. For instance, it is conceivable that health expectations and health perceptions are less important at the beginning of the sickness absence period but that these factors become more manifest during long-term absence because of growing uncertainty and an awareness or lack of future perspectives during sick leave. Therefore, in this study, we examined prognostic factors for the future work participation of medium- and long-term sick-listed unemployed and temporary agency workers with psychological problems. We aimed to identify prognostic factors that emerged during the sick leave period, in addition to unchanging prognostic factors. To achieve this, we chose to study the prognostic factors of workers who had been sick-listed for 10 and 18 months so that we could identify those sick-listed workers who are at high risk for work disability at different stages of sickness absence. The relevance for practice lies in the need for physicians or other health professionals to assess the prognosis of future work participation of those who have not returned to work within a given time-frame.

Considering the need for evidence about prognostic factors for the work participation of sick-listed unemployed and temporary agency workers with psychological problems and the great risk for work disability of these workers, further research of this underexposed group is important. Prognostic research could provide information to help identify sick-listed workers with a high-risk for work disability and provide input for sickness absence counseling.

The following research questions were formulated for unemployed and temporary agency workers who were sick-listed due to psychological problems: (1) what are the prognostic factors for work participation at 18 months among workers who had been sick-listed for 10 months (medium-term): (2) what are the prognostic factors for work participation at 27 months among workers who had been sick-listed for 18 months (long-term)?

## Methods

### Design

This study involved a longitudinal cohort survey of sick-listed unemployed workers (workers without an employment contract), temporary agency workers and fixed-term contract workers (those who had an expired contract while they were sick-listed) registered at the Dutch social security agency (SSA). The information collected for this study was part of a national survey. The cohort was followed for one and a half years, and three measurements were taken: a baseline measurement at 10 months after reporting sick (T1) and two follow up measurements at 18 (T2) and 27 (T3) months after reporting sick.

### Population

A total of 5,754 unemployed and temporary agency workers who had reported sick early December 2006-late January 2007 and had been sick-listed for 9 months received a questionnaire. Of those 2,408 entered the cohort by replying to the questionnaire. From the cohort, we selected a subset of 932 sick-listed unemployed and temporary agency workers for our study whose main reason for sickness absence were (self-reported) psychological complaints. Inclusion criteria for entry in the cohort were as follows: age between 16 and 64 years, reporting sick around January of 2007 and have been sick-listed for at least 9 months at baseline, (self-reported) mental complaints and unemployed or temporary agency worker status.

### Procedure

At follow-up, only the sick-listed workers who had responded to the previous questionnaire were approached. The questionnaires were sent to members of the study population at their home address.

### Parameters

The independent and dependent variables listed below were collected through questionnaires as used in international literature and studies [13–15] and in several epidemiological studies on work disability from 1985 till 2003 in The Netherlands [16, 17]. The potential independent prognostic variables at T1 were demographic characteristics, health characteristics (perceived health, reason for sick listing, health complaints before reporting sick), work-related factors (type of worker, RTW interventions, self-reported perceived RTW interventions from SSA), and full RTW expectations (regarding health). The potential prognostic variables at T2 were demographic characteristics, health characteristics (perceived health, reason for sick listing), work-related factors (type of worker, RTW interventions, self-reported perceived RTW interventions from SSA, work status at 18 months after reporting sick), and full RTW expectations (regarding health).

#### Independent Variables

##### Demographic Characteristics

The following demographic characteristics were determined: (a) age (in years), (b) gender (male, female), (c) marital status (married or living with a partner, single, widowed or divorced), (d) ethnicity (native Dutch versus non-native was assessed in two questions regarding the country of birth for the sick-listed person and his/her parents), and (e) educational level (low, medium or high). Low educational level included primary school, lower vocational education, and lower secondary school. Medium educational level included intermediate vocational education and upper secondary school. High educational level included upper vocational education and university.

##### Health Characteristics

The following health characteristics were determined: (a) perceived health of the sick-listed person (self-report, based on a single item: “In general, how is your state of health now?”; answer categories were: poor, moderate, good), (b) reason for sick listing ([cause of absenteeism]; self-report, single item: “What were the health complaints with which you reported sick around January 2007?”; different pre-categorized answers with physical and psychological complaints were possible, answers categories that mentioned mental distress, burn-out and other psychological complaints were selected for further analysis), and (c) health complaints before reporting sick (self-report, single item: “Did you experience health complaints before reporting sick?”; answer categories were: 0–6 months, 6–12 months or longer than 12 months before reporting sick).

##### Work-Related Factors

The following work-related factors were assessed: (a) type of worker (unemployed or temporary agency/fixed-term contract worker), (b) RTW interventions (self-report, single item: “From which agencies did you receive RTW interventions after reporting sick?”; pre-categorized answers listing different agencies, including the vocational rehabilitation agency, employment agency, occupational healthcare service, SSA, employer/temporary employment agency or other agencies), (c) the perceived efforts of RTW interventions from the SSA (self report, single item: “from 1 to 10, how do you rate SSA’s efforts to keep you working or get you back to work?”), and (d) work status of the sick-listed person 18 months after reporting sick (self-report, assessed in three questions related to work resumption since the previous questionnaire; work status [part- or full-time or sick-listed again]; and work circumstances [i.e., work adaptations, working conditions, and work hours]).

##### RTW Expectations

Regarding health, the respondent was asked whether he/she expected a full RTW in the future (self-report, single item: “do you think your health will permit a full RTW (again) in the future?”; answers categories were: yes, at the same job, yes in another field of work, I do not expect it, I do not know).

#### Dependent Variable

##### Work Participation

The outcome variable was work participation, which was measured in four questions. Work participation was operationalized as a partial or full RTW (e.g., for temporary agency workers) or the ability to work (e.g., for unemployed workers) and not being sick-listed anymore (i.e., no RTW because no employer was available). Full RTW was defined as either working the same number of hours as at the last job the respondent held before reporting sick, or as a RTW with no more sick listing. Partial RTW was defined as working fewer hours than at the last job the respondent held before reporting sick, including unpaid work, or working at another type of job while still officially sick-listed. The outcome assessments at 18 and 27 months after reporting sick were similar, with the exception of ability to work, which was not measured at 27 months. The variables we selected for this study are presented in Table 1.

**Table 1:** Overview of the selected variables and measurement moments for this study

	Prognostic variables (for both unemployed and temporary agency workers)	10 months	18 months	27 months
Category 1	Demographic factors			
	Age	*X*		
	Sex	*X*		
	Marital status	*X*		
	Ethnicity	*X*		
	Education	*X*		
Category 2	Health characteristics			
	Perceived health	*X*	*X*	
	Reason for sick listing	*X*	*X*	
	Health complaints befo re reporting sick	*X*		
Category 3	Work-related factors			
	Type of worker (unemployed/temporary agency/fixed-term contract worker)	*X*		
	RTW interventions	*X*	*X*	
	Perceived RTW intervention efforts by SSA^a^	*X*	*X*	
	Work status 18 months after reporting sick		*X*	
Category 4	RTW expectations			
	Full RTW expectation	*X*	*X*	
	Dependent variables			
	RTW (partial or full)^b^		*X*	*X*
	Ability to work^c^		*X*	*–*

^a^SSA = Dutch social security agency

^b^Full RTW (return to work) was defined as working the same number of hours as worked at the last job before reporting sick or RTW with no further sick listing; partial RTW was defined as working fewer hours than at the last job before reporting sick and included unpaid work or work at another type of job while still sick-listed

^c^Able to work: means no longer sick listed, but did not RTW because no employer was available. The cohort survey conducted 27 months after the participants first reported sick (T3) includes only data for RTW and not for ability to work

### Analysis

The variable gender (male, female) was binominal. For continuous variables, we used the median as a cut-off point whenever possible. For all categorical variables we used a percentage of approximately 50% as cut-off point whenever possible, except for some categorical variables such as ethnicity, health complaints before reporting sick and perceived RTW interventions by SSA. The following variables were dichotomized; age (<45 vs. ≥45 years); marital status (married/living with a partner vs. single/widowed/divorced); ethnicity (native vs. non-native); educational level (low vs. medium or high); perceived health of the sick-listed person (poor vs. moderate/good); reason for the sick listing (mental distress/burn-out vs. other psychological complaints); health complaints before reporting sick (no complaints vs. complaints 0–6 months prior to reporting sick/6–12 months prior to reporting sick/longer than 12 months prior to reporting sick); type of worker (unemployed worker vs. temporary agency/fixed-term contract worker); RTW interventions (yes vs. no); perceived RTW interventions from the SSA (insufficient [score 0–5] vs. sufficient [score 6–10]); work status at 18 months after reporting sick (no RTW vs. RTW [partial or full]); full RTW expectations in the future, regarding health (no expectations vs. expectations).

Two longitudinal relationships were studied to determine prognostic factors for the future work participation of medium- and long-term sick-listed unemployed and temporary agency workers with psychological problems. First, we analyzed the relationship between the independent prognostic variables at T1 and work participation (partial or full RTW or ability to work) at 18 months (T2). Second, we analyzed the relationship between the independent prognostic variables at T2 and RTW (partial or full) at 27 months (T3). The longitudinal relationships were analyzed with the backwards stepwise logistic regression analysis method.

Prior to the backwards stepwise logistic regression analysis, we performed univariate analyses (χ^2^ tests) between the independent prognostic variables and work participation. Independent variables that med the cut-off *p* value of <0.20 were selected for inclusion in the multiple regression model. Multicollinearity testing of the remaining independent prognostic variables was conducted, and multicollinearity was assumed when the tolerance was ≤0.4. A multiple logistic regression analysis with backward stepwise selection was then performed, resulting in a final model for predicting work participation. The *p* value of the prognostic variable that was retained in the model was <0.05 (Wald statistics). The Hosmer-Lemeshow test was used to assess the goodness of fit. Response analyses between T1 and T2 and between T2 and T3 were conducted by a multiple logistic regression procedure with the variables selected for inclusion in the multiple logistic regression model as independent variables and response (yes or no) as the dependent variable (*p* < 0.05, Wald statistics). Finally, we estimated the chance of work participation for unemployed and temporary agency workers when all positive prognostic factors were present or absent. All analyses were performed using the SAS software package, Version 9.1.

## Results

### Participant Characteristics

Table 2 presents the characteristics of the cohort at baseline (T1) and T2. Of the 932 participants at baseline, 476 returned the questionnaire at T2 (51% response), and 258 returned it at T3 (54% response of the participants at T2). The participants’ mean age at baseline was 42.6 years (SD 11.0 years), and the cohort consisted of 44% men and 56% women. The majority of the participants (93%) reported poor or moderate perceived (mental) health at baseline, and 60% did not expect a full RTW. At T2, 81 (18%) participants returned to part- or full-time work, and 34 participants were able to work but did not return to work because no employer was available. At the end of the follow-up at T3, 55 (21%) participants returned to work part- or full-time.

**Table 2:** Characteristics of the cohort of sick-listed unemployed and temporary agency workers 10 months (T1) and 18 months (T2) after reporting sick

Independent variables	Cohort at T1	Cohort at T2
	*n* = 932 (%)	*n* = 476 (%)
*Demographic characteristics*		
Sex		
Male	398 (44)	205 (44)
Female	514 (56)	264 (56)
Age (years)		
18–34	232 (25)	98 (21)
35–44	260 (29)	118 (25)
45–65	418 (46)	252 (54)
Marital status		
Married/living with a partner	498 (55)	264 (56)
Single/widowed/divorced	413 (45)	204 (44)
Ethnicity		
Native Dutch	617 (68)	344 (73)
Non-native^a^	294 (32)	125 (27)
Education		
Low	448 (49)	238 (51)
High (medium and higher)	458 (51)	229 (49)
*Health characteristics*		
Perceived health		
Poor	387 (42)	182 (39)
Moderate	473 (51)	248 (52)
Good	69 (7)	44 (9)
Reason for sick listing		
Only mental distress/burn-out	203 (22)	116 (24)
Mental distress/burn-out and other psychological complaints^b^	338 (36)	161 (34)
Only other psychological complaints	391 (42)	199 (42)
Health complaints before reporting sick		
Yes	762 (82)	392 (83)
No	162 (18)	81 (17)
*Work-related factors*		
Type of worker		
Unemployed worker	475 (51)	251 (53)
Temporary agency/fixed-term contract worker	457 (49)	225 (47)
RTW interventions		
Yes	542 (59)	285 (60)
No	383 (41)	188 (40)
Perceived RTW interventions by SSA		
Score 0–5	246 (33)	71 (22)
Score 6–10	491 (67)	251 (78)
Work status 18 months after reporting sick		
RTW (partial or full)	X	81 (18)
No RTW	X	382 (82)
*RTW expectations*		
Positive expectation of a full RTW		
Yes	363 (40)	141 (32)
No	542 (60)	294 (68)

*n* at T1 ranges from 737 to 932 due to missing values

*n* at T2 ranges from 322 to 476 due to missing values

^a^Non-natives: those born outside of The Netherlands or with at least one parent born outside of The Netherlands

^b^Workers sick-listed with other psychological problems may also have had mental distress/burn out

### Univariate Analysis

Univariate analysis of the relationship between the independent variables at T1 and work participation at T2 (see Table 3), and between the independent variables at T2 and work participation at T3 (see Table 4) revealed statistically significant associations (*p* < 0.05) for perceived health (moderate-to-good), type of worker (temporary agency worker), RTW interventions and full RTW expectations. Gender (female) and ethnicity (native Dutch) were significant (*p* < 0.05) during the first longitudinal relationship (T1–T2) only, whereas education (medium educational level and higher) and work status at 18 months after reporting sick (partial or full RTW) were significant only for the second longitudinal relationship (T2–T3).

**Table 3:** Univariate associations for independent variables at 10 months (T1) and work participation at 18 months (T2) in a cohort of unemployed and temporary agency workers *(n* = *476)*

Independent variables	OR	95% CI for OR	*p*
Demographic characteristics			
Female sex versus male	1.5	1.00–2.37	<0.05*
Age < 45 year versus ≥45 yr	1.3	0.87–2.00	0.19*
Married/living with a partner versus single/widowed/divorced	1.0	0.63–1.46	0.83
Native versus non-native ethnicity	1.8	1.06–3.00	0.03*
High education versus low education	1.5	0.95–2.22	0.08*
Health characteristics			
Perceived moderate-to-good health versus poor health	4.9	2.95–8.27	<0.01*
Sick-listed with mental distress/burn-out versus other psychological complaints	1.1	0.67–1.74	0.76
No health complaints before reporting sick versus health complaints	1.7	0.99–2.78	0.06*
Work-related factors			
Temporary agency/fixed-term contract worker versus unemployed worker	1.6	1.04–2.42	0.03*
RTW interventions versus no RTW intervention	1.7	1.09–2.60	0.02*
Perceived RTW interventions by SSA, Scores 6–10 vs. Scores 0–5	1.1	0.73–1.68	0.63
RTW expectations			
Positive expectation of a full RTW versus negative expectation	2.4	1.56–3.67	<0.01*

*OR* > 1 indicates a higher association with work participation (partial or full RTW or ability to work)

*OR* < 1 indicates a lower association with work participation

*OR* odds ratio, *CI* confidence interval

* Independent variables with associations for which *p* < 0.20 were selected for the multiple logistic regression analysis

**Table 4:** Univariate associations for independent variables at 18 months (T2) and work participation at 27 months (T3) in a cohort of unemployed and temporary agency workers *(n* = *258)*

Independent variables	OR	95% CI for OR	*p*
Demographic characteristics			
Female sex versus male	1.8	0.98–3.37	0.05*
Age < 45 year versus ≥45 yr	3.7	1.97–6.98	<0.01*
Married/living with a partner versus single/widowed/divorced	0.5	0.46–1.52	0.55
Native versus non-native ethnicity	1.2	0.59–2.46	0.61
High education versus low education	1.9	1.02–3.52	0.04*
Health characteristics			
Perceived moderate-to-good health versus poor health	2.7	1.30–5.75	<0.01*
Sick-listed with mental distress/burn-out versus other psychological complaints	0.9	0.43–1.68	0.65
Work-related factors			
Temporary agency/fixed-term contract worker versus unemployed worker	2.6	1.39–4.78	<0.01*
RTW interventions versus no RTW intervention	2.1	1.06–4.02	0.03*
Perceived RTW interventions by SSA, Scores 6–10 vs. Scores 0–5	1.0	0.54–1.77	0.93
Work status 18 months after reporting sick, RTW (partial or full) vs. no RTW	16.5	7.60–35.87	<0.01*
RTW expectations			
Positive expectation of a full RTW versus negative expectation	3.0	1.57–5.75	<0.01*

*OR* > 1 indicates a higher association with work participation (partial or full RTW)

*OR* < 1 indicates a lower association with work participation

*OR* odds ratio, *CI* confidence interval

* Independent variables with associations for which *p* < 0.20 were selected for the multiple logistic regression analysis

### Multiple Logistic Regression Analysis

The following variables had a cut-off *p* value of <0.20 in the univariate analysis and were selected for the multiple logistic regression analysis of the relationship between the independent variables at T1 and work participation at T2: gender, age, ethnicity, educational level, perceived health, health complaints before reporting sick, type of worker, RTW interventions and full RTW expectations (see Table 3). For the multiple logistic regression analysis between the independent variables at T2 and work participation at T3, the following variables were selected: gender, age, educational level, perceived health, type of worker, RTW interventions, full RTW expectations and work status at 18 months after reporting sick (see Table 4). The results of the multiple logistic regression analysis are presented in Table 5. The Hosmer-Lemeshow test revealed that both prediction models fit (*p* = 1.0 for the T1–T2 prediction model and *p* = 0.9 for the T2–T3 prediction model).

**Table 5:** Multiple logistic associations between independent variables and work participation in a cohort of unemployed and temporary agency workers

Independent variables (predictors)	Odds ratio	CI	*p* value
T1 → T2 backward stepwise, final model at 18 months^a^			
Perceived health (moderate-to-good)	4.2	(2.43–7.20)	<0.01
Positive expectation of a full RTW	1.7	(1.08–2.71)	0.02
T2 → T3 backward stepwise, final model at 27 months^b^			
Age <45 year	2.5	(1.10–5.70)	0.03
Work status at T2 (yes)	24.0	(8.37–69.20)	<0.01
Positive expectation of a full RTW	2.6	(1.12–5.86)	0.03

^a^Associations between independent variables at 10 months (T1) and work participation at

18 months (T2) *(n* = *476)*

^b^Associations between independent variables at 18 months (T2) and work participation at

27 months (T3) *(n* = *258)*

#### Prognostic Factors at 10 Months

The prognostic factors for sick-listed unemployed and temporary agency workers at 10 months (medium-term) for work participation at 18 months are presented in Table 5. The final analysis for prognostic factors at 10 months identified two prognostic factors for work participation: moderate-to-good perceived health (OR = 4.2) and positive full RTW expectation (OR = 1.7). At 10 months, 133 sick-listed unemployed and temporary agency workers had both moderate-to-good perceived health and a positive RTW expectation and 162 with both poor perceived health and a negative RTW expectation. The predicted chance for work participation at 18 months for the group with both positive prognostic factors at 10 months was 42, versus 9% for the group with both negative factors.

#### Prognostic Factors at 18 Months

The prognostic factors for sick-listed unemployed and temporary agency workers at 18 months (long-term) for work participation at 27 months are presented in Table 5. The final analysis for prognostic factors at 18 months identified three prognostic factors for work participation: age <45 years (OR = 2.5), work status at T2 (OR = 24.0), and positive full RTW expectation (OR = 2.6). At 18 months, 11 sick-listed unemployed and temporary agency workers were younger than 45 years, were working and had a positive RTW expectation. In contrast, 96 participants were 45 years or older, were not working and had a negative RTW expectation. The predicted chance for work participation at 27 months for the group that had all three positive prognostic factors at 18 months was 90, versus 6% for the group with all three negative factors.

### Response Analysis

There were no statistical differences between respondents and non respondents with regard to the prognostic variables at 10 months: perceived health (*p* = 0.24), full RTW expectations (*p* = 0.28). Further there were no statistical differences between respondents and non respondents with regard to the prognostic variables at 18 months: age (*p* = 0.16), work status at T2 (*p* = 0.37), full RTW expectations (*p* = 0.48). Table 6 presents the demographic variables and analysis of differences between the respondents and non respondents. Although there were statistical significant differences for some demographic variables between the respondents and non respondents, these differences do not have further implications because these variables did not remain as prognostic factors in the final model after multiple logistic regression analysis.

**Table 6:** Demographic variables of respondents and non respondents at 10 months (T1), 18 months (T2) and 27 months (T3) after reporting sick

Demographic variables	Cohort at T1, (*n* = 932)	Cohort at T2, (*n* = 476)		Cohort at T3, (*n* = 258)			Response analysis T1–T2 (*Wald statistics*)	Response analysisT2–T3 (*Wald statistics*)
		*Resp* ^*a*^	*Non resp* ^*b*^	*Resp*	*Non resp*			
Sex								
Male	398	205	193	122	86		*p* = 0.98	*p* = 0.01
Female	514	264	250	133	134			
Age								
<45 years	492	*216*	276	101	115		*p* < 0.01	*p* = 0.16
≥45 years	418	252	166	152	106			
Marital status								
Married/living with a partner	498	264	234	152	115		*p* = 0.63	*p* = 0.21
Single/widowed/divorced	413	204	209	102	105			
Ethnicity								
Native Dutch	617	344	273	192	156		*p* < 0.01	*p* = 0.65
Non-native	294	125	169	62	65			
Education								
Low	448	238	210	125	118		*p* = 0.73	*p* = 0.16
High (MBO and higher)	458	229	229	128	102			

*n* at T1 ranges from 737 to 932 due to missing values

*n* at T2 ranges from 322 to 476 due to missing values

*n* at T3 ranges from 243 to 258 due to missing values

^a^Respondents

^b^Non respondents

## Discussion

The purpose of this longitudinal cohort study was to identify prognostic factors for the future work participation of medium- and long-term sick-listed unemployed and temporary agency workers with psychological problems. Our study indicated that workers’ own perceived moderate or good health and positive expectations of a full RTW at 10 months were prognostic factors for work participation at 18 months. Younger age (<45 year), working status at 18 months (part- or full-time) and positive expectations of a full RTW at 18 months were prognostic factors for work participation at 27 months.

This study is useful because we conducted three measurements over a long period (1.5 years), so we were able to identify prognostic factors for work participation at different stages of sickness absence. This strategy revealed that the prognostic factors and their relative importance differed for medium- and long-term sickness absence. Perceived health was the strongest prognostic factor in medium-term sickness absence, whereas being at work (work status) was the strongest prognostic factor in long-term sickness absence. Furthermore, we noticed that the relative importance of full RTW expectations as a prognostic factor for work participations increased from the medium- to long-term sickness absence measurement. When conducting sickness absence counseling, occupational and insurance physicians must be aware of the change of prognostic factors and their relative importance over the course of sickness absence so they can identify high-risk sick-listed workers at different stages. Furthermore, a lack of certain positive prognostic factors (e.g., perceived good health) can provide input for the sickness absence counseling or may help the direction of RTW interventions. The practical value of the prognostic factors found in our study is clear from the 33% increased chance of work participation at 18 months when both positive prognostic factors (moderate or good perceived health and positive expectations for a full RTW) are present at 10 months. An 84% increase in the chance of work participation at 27 months was found when all three prognostic factors (age under 45 years, positive work status at T2 and positive expectations for a full RTW) were present at 18 months. However, only a small number of workers showed all three positive prognostic factors at 18 months.

To the best of our knowledge, no studies have attempted to investigate the association between prognostic factors and work participation for (long-term) sick-listed unemployed and temporary agency workers with psychological problems. Studies that have investigated the association between prognostic factors and work participation for sick-listed workers with both psychological and physical problems included only employed workers and focused mostly on the first year of sickness absence [13, 14, 18–22]. Our findings were in line with the prognostic factors reported in studies of employed workers with psychological and physical problems [13, 20, 21]. However, in contrast with these studies, we found that education, gender, health complaints before reporting sick (history of previous sickness absence) and RTW interventions were not prognostic factors for work participation for sick-listed unemployed and temporary agency workers after multiple logistic regression.

Although our study did not identify sickness absence counseling (RTW intervention) as a prognostic factor for work participation after multiple regression analysis, appropriate sickness absence counseling aimed at targeting the modifiable prognostic factors in high-risk sick-listed unemployed and temporary agency workers found in our study could help improve work participation. Perhaps the interventions used in this study were not effective for this vulnerable group because they did not target the prognostic factors found in our study. Whether sickness absence counselling aimed at targeting the modifiable prognostic factors in high-risk sick-listed unemployed and temporary agency workers found in our study actually leads to greater work participation needs to be evaluated in further research. In addition to the focus on high-risk sick-listed unemployed and temporary agency workers, sick-listed persons with existing favorable prognostic factors for work participation should be encouraged and advised to seek help to realise their potential for work participation. Furthermore, special attention should be paid to work participation (partial or full RTW) as a strong prognostic factor for future work participation. Since this vulnerable group often cannot benefit from the positive effects of (part-time) work placement because no employment is available, it would be interesting to evaluate options for sick-listed unemployed and temporary agency workers to participate in the labor market as part of a reintegration program. This approach would hopefully lead to greater work participation. A final recommendation is to include the quality and sustainability of employment in future research.

## Conclusion

We conclude that individuals’ own appraisal and assessment of their health (perceived health and RTW expectation), along with age and partial or full RTW (work status), are prognostic factors for the work participation of sick-listed unemployed and temporary agency workers with psychological problems. Furthermore, the relative importance of prognostic factors could change during long-term sickness absence. The factors found in this study may help to identify high-risk sick-listed unemployed and temporary agency workers 10 and 18 months after reporting sick. Because data on these prognostic factors are easy to collect or are already available to occupational or insurance physicians, the outcome of this study could provide input for targeted interventions aimed at sickness absence counseling and may improve work participation.
